# Adolescents’ perceived school climate, self-reported mental health, and frequency of physical activity: associations and gender differences

**DOI:** 10.3389/frcha.2025.1674304

**Published:** 2026-01-16

**Authors:** Baiba Martinsone, Eva Ģērmane, Jesslynn R. Neves-McCain

**Affiliations:** 1 Department of Psychology, University of Latvia, Riga, Latvia; 2 Department of Counseling, School Psychology and Sport, University of Massachusetts Boston, Boston, MA, United States

**Keywords:** adolescents, gender differences, mental health, physical activity, school climate

## Abstract

This study aimed to test whether a more positively perceived school climate and more frequent physical activity are related to better mental health among adolescents. Additionally, the research examined the gender differences in adolescents’ self-reported mental health, perceptions of school climate, and the frequency of their physical activity. A total of 2,526 students (52.6% girls; 43.8% boys) from grades 5 through 12 from various schools in Latvia participated in the study. The research used the Georgia School Climate Survey (GSCS) and three questions from the Physical Activity Questionnaire for Older Children (PAQ-C). The results of multiple linear regression analysis confirmed that a more positively perceived overall school climate (B = −1.05, *p* < .001) and greater engagement in physical activity (B = −1.05, *p* < .001) were both significantly associated with better mental health in adolescents. The findings also revealed gender differences in self-reported mental health: girls reported significantly higher levels of mental health difficulties (M = 2.22 compared to boys’ M = 1.87). In contrast, girls rated such factors of the school climate as adult and peer support, social/civic learning and physical environment more positively, while boys reported significantly higher levels of physical activity (28.98 compared to girls’ 26,43). Despite such limitations of the research as self-report bias and cross-sectional design, these findings underscore the critical role of the school environment in predicting mental health outcomes, highlighting the need for targeted improvements that both mitigate risk factors and promote inclusive access to physical activity among all students.

## 1 Introduction

Adolescent mental health is increasingly recognized as a significant public health concern. There is growing awareness of the need to prioritize mental health within broader public health agendas, particularly through the development of a unified action plan for promoting mental well-being across Europe (1). In Latvia, statistics for the period from 2018 to 2023 indicate that the primary reasons adolescents seek psychological support include depression, anxiety, relationship difficulties, and a lack of academic motivation (2). A recent national survey on health-related behaviors among Latvian residents reported that approximately 53% of respondents aged 15–24, across both genders, had experienced heightened tension, stress, and low mood within the past month (3). These symptoms can contribute to long-term challenges in academic achievement, social functioning, and overall health.

Mental health is defined as a state of emotional, psychological, and social well-being in which individuals are able to realize their potential, cope with everyday stressors, engage successfully in learning and productive work, build meaningful relationships, and contribute to their communities (4, 5). Mental health problems, by contrast, can cause substantial impairments in cognitive functioning, emotional regulation, and behavior, significantly impacting daily functioning (6). Failure to address mental health problems during adolescence can lead to diminished physical and psychological well-being and reduce quality of life well into adulthood (7).

Mental health challenges typically emerge as a result of the complex interplay of personal, social, and environmental factors (8). A key environmental determinant of adolescent mental health is school climate. This construct reflects the shared norms, goals, values, and attitudes that influence interpersonal relationships, teaching and learning processes, leadership practices, and institutional structures [see, e.g., (9–11)]. In this study, school climate is conceptualized as a multidimensional construct, encompassing school connectedness, peer and adult social support, cultural acceptance, social and civic learning, the physical environment, school safety, and perceptions of order and discipline (12).

A positive school climate, a supportive learning environment, teacher engagement, and parental involvement are key protective factors that help reduce adolescents’ behavioral and disciplinary challenges, improve academic and social adjustment, and thus contribute to identity development and overall mental well-being (13–19). Research indicates that the most salient contributors to adolescent depression—or exacerbations of its symptoms—include poor relationships with teachers and academic overload, both of which can lead to excessive stress and limited opportunities for rest and recreation (20). Among the most significant school climate factors associated with mental health are peer relationships, teacher-student relationships, and a sense of school connectedness (21). Positive interactions between students and teachers are known to reduce behavioral and mental health problems, enhance adolescents’ self-perception, increase their engagement in school life, and strengthen their sense of belonging (10). Findings from an international study confirmed that a positive school climate is associated with more favorable mental health indicators among adolescents (9). A longitudinal study examining various dimensions of mental health also found that a positively perceived school climate predicted stronger school identification, greater happiness, increased life satisfaction, and more positive emotional states (22). These results support the understanding of school climate and connectedness as factors that contribute to adolescents’ mental health; however, some contradictory findings have also emerged. For example, a longitudinal study conducted in Sweden reported no significant correlation between school climate and mental health among adolescent participants, though such an association was observed in the teacher sample (23). This highlights the importance of continued research into the relationship between school climate and mental health to gain a more nuanced understanding of how these constructs interact.

In 2024, the European Commission issued recommendations to enhance student well-being in schools (24, 25). These recommendations emphasize a whole-school approach to fostering a positive school climate and implementing social-emotional education. They further underscore the importance of safeguarding fundamental rights by promoting core enablers of well-being, such as spending time outdoors and incorporating physical activity and sports into the school curriculum. The United Nations has also developed comprehensive standards and guidelines to implement its vision “Every School a Health-Promoting School” (4). These initiatives aim to integrate and enhance physical and mental health programs within school systems to promote student well-being.

However, it is increasingly observed that such active outdoor behaviors as running, playing sports, and cycling are often being replaced by sedentary activities like screen time and gaming. Such behavioral shifts can have detrimental effects on children's and adolescents’ physical and mental health [see, e.g., (26)]. Therefore, it is essential to introduce or reinforce healthy lifestyle habits during adolescence, as the physical, cognitive, and socio-emotional resources developed during this stage form the foundation for achieving better health and well-being later in life (27). One of the most significant factors supporting and promoting mental health is regular physical activity, which can positively influence an individual's psychological functioning, personality development, and overall quality of life (28). Research shows that engaging in weekly physical activity of varying intensity levels can be beneficial in alleviating mental health difficulties, including among children with ADHD symptoms (29). Accordingly, it is recommended that children and adolescents aged 5–17 years (including those with disabilities) engage in at least 60 min of moderate to vigorous aerobic physical activity daily (30). A systematic review shows that moderate-to-vigorous, especially aerobic, physical activity significantly improves key mental health outcomes, including subjective well-being, self-esteem, and emotional self-regulation (31). Studies also demonstrate that adolescents who engage in physical activity two to five times per week report lower levels of stress, anger, and depressive mood compared to those who are inactive (32, 33), as well as reduced anxiety symptoms (34). Physical activity has also been shown to strengthen self-awareness, foster a more positive self-concept and confidence in one's abilities (35), enhance academic performance (36), improve social integration (32), foster mental well-being (37), and predict stronger emotional self-regulation skills, higher self-esteem, greater life satisfaction, and lower levels of anxiety and depression among adolescents (38).

It is well established that the social environment plays a critical role in shaping students’ physical activity behaviors. Among reasons why students choose to be physically active is the enjoyment of spending quality time with others, which in turn fosters social bonds and emotional closeness. Additionally, the school physical environments can affect students’ willingness to engage in sports and exercise. Conversely, certain social factors serve as barriers to physical activity, including peer bullying, inadequately maintained school facilities, and prevailing attitudes among parents and school staff that prioritize academic achievement over participation in sports (39).

Studies have identified gender differences in the evaluation of school climate; however, findings remain inconsistent. Some research indicates that girls report a more positive perception of the school climate than boys [see, e.g., (40)], while other studies suggest that girls actually rate the school climate less favorably than boys [see, e.g., (41)]. Similarly, gender disparities are evident in mental health indicators. Over the past two decades, depression rates have risen globally, particularly in high-income countries, with the most significant increase observed among adolescent girls aged 10–19. In contrast, boys are more likely to exhibit externalizing behavioral problems, although recent trends also show a rise in internalized distress among boys (42). With regard to physical activity, studies consistently show that boys engage more frequently in active physical pursuits and display greater diversity in the types of physical activities they undertake (43). Conversely, participation in physical activity among girls has been linked to better academic performance (44, 45). However, limited research exists on these interactions in Eastern European contexts.

The present study focuses on examining the relationships between adolescents’ perceived school climate, the frequency of physical activity, and self-reported mental health, with particular attention paid to gender differences. The study is guided by the following research questions and hypotheses:

RQ1: What is the relationship between student perceptions of school climate and self-reported physical activity with respect to mental health ratings?

H1_A_: Adolescent self-reports of a better overall school climate perceptions and more often physical activity will significantly predict fewer mental health symptoms.

H1_B_: Relationships between school climate ratings and mental health will differ across subscales.

RQ2: Are there gender differences in self-reported mental health, perceived school climate, and frequency of physical activity?

H2: Adolescent ratings of mental health, perceived school climate, and frequency of physical activity will differ by gender.

## 2 Method

### 2.1 Participants

A total of 2,526 students participated in the study, with 2,436 valid responses included for further data analysis (52.6% girls; 43.8% boys). Students were asked to agree to participate at the start of the survey, and informed that they can skip items or discontinue the survey at any time. Only surveys with complete responses for at least one scale were included in the analyses (e.g., 90 omitted). All participants were students from grades 5 through 12, representing various lower and upper secondary schools across Latvia. The majority of participants were from lower secondary school (grades 5–9), while the fewest respondents were from upper secondary school (grades 10–12). Table 1 illustrates the participant demographics.

**Table 1:** Demographic characteristics of the sample.

Sample	*N*	*%*
Total sample	2,526	
Gender		
Girls	1,329	52.6%
Boys	1,107	43.8%
Grade		
Grade 5	347	13.3%
Grade 6	462	17.7%
Grade 7	410	15.8%
Grade 8	293	11.3%
Grade 9	400	15.4%
Grade 10	265	10.2%
Grade 11	229	8.8%
Grade 12	120	4.6%

### 2.2 Measures

Perceived school climate was assessed using the Georgia School Climate Survey (GSCS) (46). The survey consists of 36 statements rated on a 4-point Likert scale: 1—strongly disagree, 2—somewhat disagree, 3—somewhat agree, 4—strongly agree. Higher scores indicate a more positive perception of the school climate. The instrument evaluates students’ perceptions across eight dimensions: sense of school belonging, peer social support, adult social support, cultural acceptance, social/civic learning, physical school environment, school safety, and order and discipline (12).

To assess mental health, the study employed the Mental Health Subscale from the Georgia Student Health Survey. This subscale measures the frequency of depressive symptoms or other indicators of emotional dysregulation experienced over the previous 30 days. Responses were recorded on a 7-point Likert scale: 1—not at all; 2–1–2 days; 3–3–5 days; 4–6–9 days; 5–10–19 days; 6–20–29 days; and 7—all 30 days. Higher scores reflect more severe mental health difficulties, whereas lower scores suggest that the student experienced few or no mental health problems in the previous 30 days.

The frequency of respondents’ physical activity was evaluated using three items from the Physical Activity Questionnaire for Older Children (PAQ-C) (47). These items assessed whether, and how frequently, students engaged in physical activity each weekday, both during and outside of school hours. Responses were given on a 5-point Likert scale: 0—not at all, 1—a little, 2—moderately, 3—a lot, 4—very much. Higher scores reflect more frequent and intense engagement in physical activities. The internal consistency of all scales was acceptable (see Table 2).

**Table 2:** Descriptive and inferential statistics for perceived school climate, mental health, and frequency of physical activity (*N* = 2,493).

Variable	M	SD	K-S	α
Overall perceived school climate	2.93	0.45	0.05	0.91
School connectedness	2.83	0.62	0.12	0.79
Peer social support	3.33	0.63	0.18	0.66
Adult social support	3.27	0.60	0.15	0.87
Cultural acceptance	3.02	0.60	0.10	0.87
Social/civic learning	3.10	0.58	0.10	0.85
Physical environment	2.82	0.74	0.13	0.71
School safety	2.93	0.61	0.12	0.77
Order and discipline	2.17	0.65	0.12	0.67
Mental health	2.08	1.19	0.18	0.89
Frequency of physical activity	27.58	7.35	0.05	0.84

### 2.3 Procedure

Based on convenience through school connections, school principals and academic coordinators from upper and lower secondary schools across Latvia were contacted via email or phone to request their consent and support for the study to be conducted at their institutions. Using the internal electronic platform E-class or other official school communication systems, participating institutions sent informed consent forms to parents. After receiving their consent, they distributed the survey to the target student groups. In some schools, data collection was carried out in person by the first two authors of the research, with students completing paper-based questionnaires. The introduction to the questionnaire explained the purpose of the study, ensured participant anonymity, and emphasized the voluntary nature of participation before obtaining the students’ informed consent.

Data collection took place during the spring and autumn of 2024 and continued through the end of January 2025. The study received ethical approval from the Ethics Committee for Humanities and Social Sciences Research at the University of Latvia.

### 2.4 Data analysis

The research employed a correlational study design to explore the two research questions. Data analysis was performed using IBM SPSS Statistics version 29.0. Preliminary analysis included examining the data distribution for normality (e.g., Kolmogorov–Smirnov values), calculating means, standard deviations, Pearson correlation, and Cronbach's alpha values (see Table 2).

For RQ1, a multiple linear regression model was tested with overall school climate ratings and physical activity as predictors of mental health. Then, a multiple regression model was tested with school climate subscale ratings and physical activity as predictors of mental health. Both models were evaluated in terms of fit, variance explained in mental health ratings, and regression coefficients for school climate and physical activity.

For RQ2, one-way multivariate analysis of variance (MANOVA) was performed with gender (girls and boys) as an independent variable and school climate subscales, physical activity, and mental health as dependent variables. Model indices and comparison F-values were examined.

## 3 Results

The findings indicate that, overall, the participating adolescents rate the school climate positively (M = 2.93; SD = 0.45), with a tendency toward higher ratings. This is supported by a negative skewness coefficient (−0.79) and moderate kurtosis (1.56). Mental health difficulty ratings were also generally low, suggesting good overall mental health among the participants (M = 2.08; SD = 1.19). However, a high positive skewness (1.59) and elevated kurtosis (2.38) indicate considerable variability in responses—while the majority of adolescents reported infrequent experiences of mental health difficulties, some exhibited markedly high scores, suggesting frequent and severe emotional challenges. This is consistent with results using the mental health data in previous studies, indicating a typical pattern in the nature of student mental health reports (9). To address data non-normality, a nonparametric bootstrapping method with 1000 iterations was used, for all models producing a 95% boostrap confidence interval (48).

When adolescents assessed the frequency of their physical activity across weekdays, the total possible score was 28. The results (M = 27.58; SD = 7.35) suggest a generally high self-reported level of physical activity. Skewness (−0.13) and kurtosis (−0.40) values suggest a distribution close to normal, with relatively consistent activity levels across the sample.

### 3.1 Research question 1

To test the hypothesis that a more positively perceived school climate and higher frequency of physical activity would correlate with and predict better mental health in the sample of adolescents, Pearson correlation coefficients were calculated (see Table 3).

**Table 3:** Pearson correlation coefficients between adolescents’ perceived school climate, its dimensions, regular physical activity, and mental health (*N* = 2,343).

Variable	Mental health	Physical activity
Overall perceived school climate	−0.31**	0.04
School connectedness	−0.39**	0.13**
Peer social support	−0.20**	0.05*
Adult social support	−0.20**	0.04
Cultural acceptance	−0.29**	−0.05*
Social/civic learning	−0.35**	0.03
Physical environment	−0.31**	0.02
School safety	−0.21**	0.03
Order and discipline	0.24**	−0.03
Mental health	—	−0.07**

* *p* < 0.05.

** *p* < 0.01.

Given the multiple significant relationships between variables of interest, two multiple linear regression analyses were performed. In Model 1, mental health was entered as the dependent variable, while perceived overall school climate and frequency of physical activity served as independent variables. In Model 2, mental health was entered as the dependent variable, while school climate subscale ratings and frequency of physical activity served as independent variables.

The results indicate that Model 1 is statistically significant and predicts 16.8% of the variance in mental health [*F*(2, 279,82) = 237.71, *p* < .001]. A more positively perceived overall school climate (B = −1.05, *p* < .001) and more frequent physical activity (B = −0.05, *p* < .001) are associated with fewer mental health problems. However, the effects of physical activity were relatively small (partial r^2^ = .00). These results were consistent with Hypothesis 2a.

The researchers then examined which subdomains of school climate were associated with mental health. The results indicate that Model 2 is statistically significant and predicts 24% of the variance in mental health [*F*(9, 89,81) = 83.61, *p* < .001]. Consistent with Hypothesis 2b, four out of eight school climate subscales emerged as significant predictors of mental health: school connectedness (B = −0.51, *p* < .001, partial r^2^ = .05), adult social support, (B = −0.24, *p* < .001, partial r^2^ = .02), social/civic learning (B = −0.21, *p* < .001, partial r^2^ = .01), and school safety (B = −0.39, *p* < .001, partial r^2^ = .07). For each of these, positive perceptions were associated with fewer mental health problems. Model results and coefficients are presented in Tables 4, 5.

**Table 4:** Results for regression models 1 and 2.

Model	Sum of squares	df	Mean square	F	Adj. R^2^
Model 1					
Regression	559.65	2	279.83	237.71	0.17***
Residual	2,754.56	2,340	1.18		
Total	3,314.21	2,342			
Model 2					
Regression	808.25	9	89.81	83.61	0.24***
Residual	2,505.96	2,333	1.07		
Total	3,314.21	2,342			

*** *p* < 0.001.

**Table 5:** Model coefficients.

Model	Unstandardized coefficient (B)	Standard error	95% confidence intervals		*t*	Partial *r^2^*
Model 1			Lower	Upper		
Constant	5.40***	.21	5.02	5.82	32.18	
School climate	−1.05***	.06	−1.17	−.94	−21.21	.16
Frequency of physical activity	−.05*	.03	−.10	.01	−2.11	.00
Model 2						
Constant	5.38***	.22	4.95	5.80	30.29	
School connectedness	−.51***	.05	−.62	−.41	−10.96	.05
Peer social support	−.08	.06	−=.19	.04	−1.80	
Adult social support	−.24***	.05	−.33	−.15	−6.28	.02
Cultural acceptance	−.07	.05	−.17	.03	−1.27	
Social/civic learning	.21***	.06	.09	.32	4.76	.01
Physical environment	.03	.05	−.06	.12	0.70	
School safety	−.39***	.04	−.47	−.31	−12.62	.07
Order and discipline	−.06	.06	−.17	.06	−.87	
Frequency of physical activity	−.04	.03	−.09	.03	−1.67	

* *p* < 0.05.

*** *p* < 0.001.

### 3.2 Research question 2

To address gender differences in self-reported mental health, perceived school climate, its subdimensions, and the frequency of physical activity, a one-way MANOVA was performed. The results indicated that school climate subscale ratings, frequency of physical activity, and mental health differed based on gender [*F*(10, 2,253) = 27.08, *p* < .001; Pillai's trace = 0.11]. The results of the one-way MANOVA also indicated that ratings on specific mental health items were significantly different depending on gender [*F*(8, 2,263) = 38.3, *p* < .001; Pillai's trace = 0.12]. All mean comparisons are provided in Table 6.

**Table 6:** Gender differences in mental health symptoms, school climate subscale ratings, and frequency of physical activity.

Variable	Girls M (SD)	Boys M (SD)	Mean *Δ* [95% CI]	*F*	Partial *η*^2^
Mental health	*N* = 1,247	*N* = 1,034			
Overall mental health problems	2.22 (1.17)	1.87 (1.13)	.36 [.26–.45]	53.21***	.02
Feeling sad/withdrawn	3.21 (1.69)	2.63 (1.79)	.59 [.45–.73]	64.79***	.03
Sudden fear with physical symptoms	2.46 (1.62)	1.85 (1.46)	.60 [.48–.73]	85.29***	.04
Impulsive/uncontrolled behavior	1.46 (1.06)	1.63 (1.34)	.16 [.06–.26]	10.48***	.00
Eating problems	1.71 (1.46)	1.50 (1.34)	.22 [.10–.33]	13.16***	.01
Anxiety/worry	2.60 (1.77)	1.90 (1.51)	.70 [.56–.84]	101.50***	.04
Difficulty concentrating	2.01 (1.58)	1.91 (1.56)	.10 [-.03–.23]	2.27	
Mood swings	2.43 (1.75)	1.82 (1.41)	.61[.48–.75]	82.32***	.04
Extreme behavioral/personality changes	1.87 (1.42)	1.73 (1.41)	.14 [.02–.26]	5.61	
Perceived school climate	*N* = 1,312	*N* = 1,099			
School connectedness	2.86 (0.61)	2.82 (0.62)	.38 [-.01-.09]	2.19	
Peer social support	3.42 (0.59)	3.26 (0.62)	.08 [.03–.13]	9.59**	.00
Adult social support	3.38 (0.54)	3.19 (0.60)	.06 [.00–.12]	4.02*	.00
Cultural acceptance	3.08 (0.55)	2.98 (0.62)	.00 [-.05–.06]	0.01	
Social/civic learning	3.14 (0.55)	3.09 (0.59)	.19 [.15–.24]	71.23***	.03
Physical environment	2.84 (0.72)	2.84 (0.73)	.13 [.07–.18]	21.96***	.01
School safety	2.99 (0.56)	2.90 (0.65)	.01 [-.06-.07]	0.04	
Order and discipline	2.17 (0.62)	2.16 (0.69)	.09 [.04–.14]	11.69***	.00
Physical activity	*N* = 1,237	*N* = 1,027			
Frequency of physical activity	26.43 (6.84)	28.98 (7.55)	.33 [.25–.40]	74.53***	.03

* *p* < 0.05.

** *p* < 0.01.

*** *p* < 0.001.

The findings indicate that girls reported significantly higher levels of mental health difficulties (M*_Δ_* = .36, Partial *η*^2^ = .02), suggesting that girls are more likely to report symptoms of psychological distress. Girls showed significantly higher scores across multiple symptom categories, including sadness/withdrawal, sudden fear with somatic reactions, anxiety interfering with daily functioning, mood swings, and eating problems.

In contrast, boys scored significantly higher on symptoms related to impulsive and uncontrolled behavior, which may involve risks of harm to self or others. No significant gender differences were found in difficulties with concentration or restlessness.

The mean values obtained from the school climate survey indicate that girls rated the overall school climate significantly more positively than boys. Statistically significant gender differences were also found in several dimensions of the school climate, particularly in its social-emotional aspects. Girls scored significantly higher on dimensions such as peer and adult social support, social/civic learning, and perceived physical environment at school.

Gender differences in the adult and peer social support subscales suggest that girls and boys perceive the levels of support in the school environment quite differently. In contrast, both genders reported similar levels of perceived belonging to school, school safety, and order/discipline.

Furthermore, the results reveal statistically significant gender differences in the frequency of physical activity, with boys engaging in physical activity significantly more often than girls.

## 4 Discussion

Adolescence is recognized as a challenging developmental period, making it essential to examine the factors that contribute to a safer, more supportive, and healthier transition through this stage. Among the contextual factors influencing students’ development, the school environment plays a critical role—it can both help prevent mental health difficulties and serve as a key source of support when such challenges arise. Schools can positively impact adolescents by promoting social-emotional learning, encouraging healthy decision-making, and fostering behaviors that support overall well-being, such as participation in physical activity both during lessons and outside of school settings.

### 4.1 Relationship between school climate, mental health, and physical activity

The study aimed to investigate the relationship between students’ perceptions of school climate and self-reported physical activity with respect to mental health ratings. The findings confirmed that school climate is a significant predictor of adolescent mental health: a more positively perceived school climate is strongly associated with better self-reported mental health among adolescents. This aligns with findings from previous studies that show that students who report a stronger connection to their school and value other aspects of a positive school climate also tend to experience better mental health [see, e.g., (9, 49)]. The relationship between school climate and mental health is bidirectional. Mental health influences how adolescents perceive their school environment, and vice versa—students with a negative perception of school climate are more likely to experience mental health problems such as depression and anxiety (15). The role of school staff is particularly emphasized as a source of emotional support and protection for students struggling with mental health difficulties (15, 50).

The current study also demonstrated that nearly all dimensions of school climate are significantly associated with adolescent mental health. These include a sense of school belonging, indicating that students feel accepted and valued; social and civic learning, reflected in values such as fairness, helping behavior, politeness, and openness to diverse viewpoints; and cultural acceptance, which underscores the importance of respectful and equitable treatment across cultural and individual differences. The physical environment and safety at school were key factors, as was the quality of peer and adult relationships, particularly when students feel they are treated fairly and with respect by teachers and classmates. The more positively students evaluated these aspects, the fewer mental health challenges they reported. Specifically, school connectedness, adult social support, social/civic learning, and school safety emerged as significant predictors of adolescents’ mental health. These findings align with Bronfenbrenner's bioecological theory of development [as cited in (11)], which emphasizes the importance of environmental context in child and adolescent development. A school environment that fosters support, safety, and engagement can serve as a protective setting that promotes student well-being.

Interestingly, higher ratings of order and discipline at school were associated in this study with higher levels of self-reported mental health issues among adolescents. This finding contradicts evidence that adolescents who perceive the school environment as structured and orderly tend to report fewer symptoms of depression, loneliness, hopelessness, and anxiety (51). A potential explanation that requests qualitative follow-up could be that respondents misinterpreted items like “I feel my school has high standards for achievement” as too strict and demanding. Previous research suggests that heightened control and a rigid, autonomy-restricting school environment can undermine psychological well-being (52), while an overly structured, inflexible school climate may create psychological pressure and reduce students’ sense of autonomy (53).

Adolescents who were more physically active also reported better mental health. This aligns with prior research showing that regular physical activity enhances quality of life. However, the extent to which physical activity can mitigate more severe mental health issues remains inconclusive (54). Nonetheless, physical activity is recognized as an effective means of alleviating symptoms of emotional dysregulation, such as depression (55), and improving stress management (56). Physically active adolescents generally exhibit higher self-esteem, which enhances psychological resilience and coping capacity (57). Regular physical activity also promotes healthy behavioral patterns and improves overall quality of life (28).

While no association was found between overall physical activity frequency and general perceptions of school climate, significant positive correlations emerged between physical activity and two specific dimensions of the school climate: a sense of belonging and peer social support. Adolescents who felt a stronger sense of belonging and greater peer acceptance were more likely to engage in physical activity. These findings support previous research highlighting the mediating role of school climate in promoting physical activity among adolescents, particularly through peer and teacher support and a sense of safety and inclusion (16, 58, 59). However, in this sample of adolescents, self-reported lower cultural acceptance was associated with higher ratings of physical activity, which could be interpreted as a coping strategy.

### 4.2 Gender differences in perceptions of school climate, mental health, and physical activity

It was found that ratings concerning school climate, mental health, and frequency of physical activity differed based on gender. Girls were more likely than boys to report mental health challenges, including feelings of sadness and withdrawal, sudden fear with somatic symptoms, eating disorders, anxiety and worry that interfere with daily life, and mood swings. Boys, in contrast, more frequently reported externalized behavioral problems such as impulsivity and uncontrolled behavior that includes a risk of harm to self or others. These results are consistent with findings from previous studies indicating that adolescent girls are more prone to internalizing symptoms such as anxiety and depression (60). Previous research findings also confirm that girls report significantly higher rates of mental health difficulties than boys (61), with such difficulties tending to persist or even increase with age, particularly during periods of major physical, emotional, or social change (62). Boys, by contrast, tend to exhibit more externalized behaviors, such as aggression or substance use (42).

Both genders evaluated certain aspects—such as a sense of belonging, cultural acceptance, and school safety—similarly. A positive evaluation of these elements provides a strong foundation for promoting adolescent emotional well-being and fostering positive behavioral development (16). Given the study context, these findings underscore the value of a safe and inclusive school environment as a vital resource for both boys and girls.

Finally, the study revealed that boys were significantly more likely to engage in physical activity than girls, confirming a pattern from previous research (59). This difference may help explain the earlier finding that girls experience more mental health difficulties, as boys, who are more physically active, reported fewer mental health issues. This relationship supports the evidence that physical activity plays a key role in improving mental health through both biological mechanisms (e.g., release of mood-enhancing neurochemicals during exercise) and psycho-emotional benefits, such as improved self-esteem, well-being, and emotional regulation (28).

Overall, this study supports the recommendation that schools should ensure students achieve at least 60 min of daily physical activity by integrating movement across lessons, providing accessible extracurricular sports, and expanding opportunities for nature-based activities that accommodate diverse abilities and needs. Additionally, all teachers should receive targeted training that equips them to incorporate movement-based strategies into everyday instruction and to actively promote physical activity throughout the school day.

## 5 Conclusions and limitations

School climate was found to be the strongest and most significant predictor of adolescents’ mental health. The findings also confirm that adolescents who are more frequently physically active tend to report better mental health outcomes, and that girls are more likely to experience mental health difficulties than boys. Girls more frequently reported internalized symptoms and boys were more likely to report externalizing difficulties.

This research indicated gender differences in perceptions of school climate, with girls rating it significantly more positively than boys. These findings suggest that in this sample, girls may feel more emotionally safe and supported at school, reporting greater support from both peers and adults, including teachers and school staff.

This study has some limitations. Firstly, a non-random sampling approach was used to select the participants. It does not allow for generalization the conclusions taking into account potential selection bias. Secondly, all data were obtained through students’ self-reports, which are inherently subjective. Due to this reason, the physical activity was measured rather generally, addressing it's regularity but not such specific aspects as it's intensity and duration. To gain more in-depth understanding of the role of physical activity, it should be measured more detailed in the future research. Thirdly, there may be other uncontrolled variables predicting adolescents’ mental health—such as family dynamics, individual academic achievement, developmental disorders, and personality traits—that could have impacted the study outcomes, particularly in assessing both mental health and school climate. Fourthly, the respondents in the study were in grades 5 through 12, and developmental stages differ substantially between a fifth-grader and a twelfth-grader. For a more nuanced understanding, it would be useful to address the age heterogeneity and differentiate between the distinct phases of adolescence or by grade level.

Future research should implement a mixed-methods approach to gain deeper insights into how adolescents interpret school climate factors, how they experience and perceive belonging, support, and safety, and broaden the scope of mental health issues by clarifying their intensity and related aspects. While boys reported a similar sense of school connectedness to girls, it would be particularly meaningful to further explore boys’ well-being in the school context, including the availability of social support and access to assistance from school personnel. A qualitative inquiry would also help to better understand the adolescents who report low physical activity levels by identifying the alternative ways they spend their time and understanding the barriers that prevent them from engaging in physical activity more frequently.

## Data Availability

The raw data supporting the conclusions of this article will be made available by the authors, without undue reservation.
